# Experimental Study on Ultrasonic Assisted Turning of GH4068 Superalloy

**DOI:** 10.3390/ma16093554

**Published:** 2023-05-05

**Authors:** Renke Kang, Pengnan Zhang, Zhaocheng Wei, Zhigang Dong, Yidan Wang

**Affiliations:** State Key Laboratory of High-Performance Precision Manufacturing, School of Mechanical Engineering, Dalian University of Technology, Dalian 116014, China; kangrk@dlut.edu.cn (R.K.); zpengnan@163.com (P.Z.); wei_zhaocheng@dlut.edu.cn (Z.W.);

**Keywords:** GH4068 superalloy, ultrasonic assisted turning, cutting force, surface morphology

## Abstract

GH4068 superalloy is a new type of nickel-based superalloy in the aerospace field. It is an important alloy material for the manufacture of aircraft tubular components and aero-engine hot-end components. These components need to be machined with good surface quality to meet their use requirements. New hybrid machining processes can improve the quality of surface finish compared to conventional machines. In this paper, ultrasonic assisted turning (UAT) technology was applied to the machining of GH4068 superalloy. The experimental system of UAT was established. Experiments of UAT and conventional turning (CT) of GH4068 superalloy were carried out to study the effects of cutting speed, feed speed, cutting depth and vibration amplitude on cutting force and surface roughness. The surface morphology of the workpiece and chip were observed. The experimental results show that *F*_x_ and *F*_y_ can be reduced by a maximum of 44% and 63%, respectively, and the surface roughness can be reduced by a maximum of 31% after adding ultrasonic vibration. Compared with CT, the UAT has a better machining quality, a more obvious chip-breaking effect, and a smaller chip bending radius, which guides the high-quality processing of the GH4068 superalloy.

## 1. Introduction

Superalloy is an austenitic metal material based on iron, nickel, and cobalt, which can withstand high strength and complex stress above 600 °C. Superalloys have been widely used in aerospace, shipbuilding, automotive and biomedical fields due to their excellent high-temperature strength, mechanical and chemical properties, high corrosion resistance, high melting point, and thermal fatigue resistance [1,2,3,4]. As indicated by statistics, nickel-based superalloys account for more than half of the aerospace industrial materials [5], and the proportion is growing steadily. GH4068 is a new type of nickel-based superalloy, which combines the advantages of nickel-based and cobalt-based superalloys. It increases the content of Co and Ti in the alloy design, strengthens the medium-temperature service zone of the alloy material, weakens the high-temperature processing zone, which ensures that the alloy has sufficient strength in the working temperature zone (yield strength ≥ 1500 MPa at 750 °C), and improves the hot workability (elongation > 1000% at temperature > 950 °C). It is an important alloy material applied to the new generation of aircraft tubular components and aero-engine hot-end components [6,7]. The complex parts made by GH4068 usually include rotary surfaces such as shafts, head cones, and nose cones, which need to be turning precisely. GH4068 superalloy has stable internal structure, small thermal conductivity and strong plastic deformation ability. In the actual processing process, the traditional turning processing method has poor performance, which exposes a series of problems, including the large cutting force, the high cutting temperature, the severe tool wear, the poor quality of processing surface, and the uncontrollability of machining accuracy [8].

UAT is an effective processing method for difficult-to-machine materials such as composite materials, titanium alloys, and high-strength steels. In the process of cutting, the addition of ultrasonic vibration changes the contact cutting state of the tool and the material, and the continuous processing of CT is changed into intermittent processing by the cutting method of periodic separation of the tool and the workpiece [9,10]. Scholars have expounded on the advantages of UAT from the aspects of cutting force, surface quality, and chip morphology. Moriwaki et al. [11] used a diamond cutter for ultrasonic turning of stainless steel and found that intermittent cutting helped reduce the cutting force. Liu et al. [12] analyzed the cutting force of diamond tool UAT and CT SiCp/Al composites and concluded that the main cutting force of UAT is much lower than that of CT. The influence of cutting speed on cutting force is the most obvious, and the influence of feed speed is the least. Xu et al. [10] conducted a comparative experiment of turning 304 austenitic stainless steel with or without ultrasonic vibration. The results show that there is an optimal amplitude range for ultrasonic turning to reduce cutting force. Among the cutting parameters, the cutting depth has the greatest influence on the cutting force, followed by the cutting speed and feed rate. Chen et al. [13] systematically studied the influence of UAT on cutting performance in different scenarios, and proposed a new model to determine the average cutting force and transient cutting force. Maurotto et al. [14] compared the surface texture of UAT and CT titanium alloy and observed that the surface of CT has obvious periodicity compared with UAT. At the same time, the surface roughness level is calculated. Compared with CT, the surface roughness of UAT is reduced by 49%. Lotfi et al. [15] analyzed the 3D surface morphology of UAT AISI4140 steel. Due to the lower radial force in the ultrasonic machining process and the longer separation time between the tool and the chip, the surface roughness is also improved significantly. Yan et al. [16] found that long spiral chips were produced during the ordinary turning of titanium alloys. The chips produced by ultrasonic turning are segmented due to ultrasonic vibration. This kind of chip is thinner and smaller and has less influence on the surface roughness of the workpiece. Xu et al. [17] found that the chip deformation generated by CT is serious, as the surface is rough, having defects including grooves, scratches, protrusions and so on. The chip deformation generated by UAT is relatively small, the chip-breaking effect is ideal, the surface is smoother, and regular ultrasonic vibration traces can be observed. In summary, methods of UAT changes the removal mechanism of the material, reduces the cutting force and tool wear, and improves the machining quality.

Similarly, UAT has also been used in the processing of superalloys in recent years. Wei et al. [18] conducted experimental studies on UAT and CT of two superalloys Inconel 718 and 625. It was found that the surface grooves of the processed Inconel 625 have a larger spacing and a better surface morphology than Inconel 718. Jay et al. [19] demonstrated that UAT produces thinner and shorter chips than CT when machining Nimonic 90 superalloy with UAT and CT. Chandra Nath et al. [20] found that the workpiece-tool contact ratio is the key factor to determine the cutting force. Through the cutting experiment of Inconel 718 superalloy, it is found that ultrasonic turning can obtain a higher machining quality and reduce the cutting force significantly at a lower cutting speed. Babitsky et al. [21] compared the surface roughness and roundness of Inconel 718 superalloy in UAT and CT under the same parameters. For the samples processed by ultrasonic turning, Ra is increased by about 50%, the regularity of the surface contour is greatly improved, and the roundness of the workpiece is also greatly improved.

Airao et al. [22] conducted a comparative study of the machinability of Ti-6Al-4V and Nimonic 90 under CT and UAT, which significantly reduced energy consumption and improved tool life of both materials. Nirala et al. [23] combined ultrasonic vibration with lubrication (MQL) and cooling (LCO2) to improve the workability of Inconel 718. Compared with the widely studied Inconel 718 and Nimonic 90 superalloy, the newly developed superalloy GH4068 has higher strength and more difficult processing. UAT is also expected to become a method to improve processing quality. For GH4068 superalloy, the main research focuses on material preparation and material properties. In the study of Wang et al. [24], friction stir processing (FSP) was for the first time performed to high-strength and high-melting-point Ni-Co based superalloy (GH4068), and enhanced strength and ductility were achieved in FSP samples. Liu et al. [25] studied the hot deformation behavior and machinability of GH4068 superalloy. However, there are no literature reports on the turning of GH4068 superalloy. In this paper, single-factor experiments of UAT and CT GH4068 superalloy were carried out in this paper to explore the influence of process parameters such as cutting speed, feed speed, cutting depth, and vibration amplitude on cutting force and surface roughness. The surface morphology and chip morphology characteristics in CT and UAT were observed and the optimal processing parameters are determined, which provided a reference for the high-quality processing of GH4068 superalloy.

## 2. Materials and Methods

### 2.1. Tools and Materials

**Figure 1:**
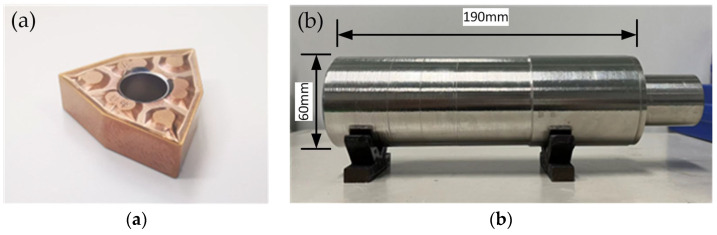
Tool and material: (**a**) WNMG080404-TF blade, (**b**) GH4068 superalloy.

In this experiment, WNMG080404-TF-coated cemented carbide blades (XinLi, Dalian, China) were used as shown in Figure 1a. Considering the tool needs to have sufficient strength to reduce the impact on the experimental results. Therefore, the geometrical parameters of tool are shown in Table 1.

The workpiece used in the experiment is GH4068 superalloy as shown in Figure 1b. This stepped cylindrical workpiece has a diameter of 60 mm and a length of 190 mm for its large end.

The chemical composition of the workpiece material is shown in Table 2.

### 2.2. Machining System of UAT

**Figure 2:**
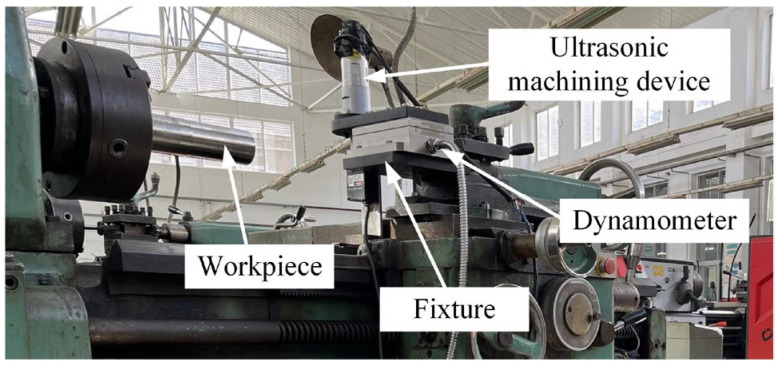
Experimental device of Ultrasonic turning.

The UAT experimental device is shown in Figure 2. The experiment of UAT superalloy is carried out on the CA6140A lathe (Dalian Machine Tool Group Corp, Dalian, China), with the highest spindle speed being 1400 r/min. In the experiment, the workpiece clamped by the three-jaw chuck rotates at high speed, which is the main motion of cutting. The tool is installed at the front end of the vibration system and fixed with the skateboard of the machine tool to realize the feed movement of cutting.

**Figure 3:**
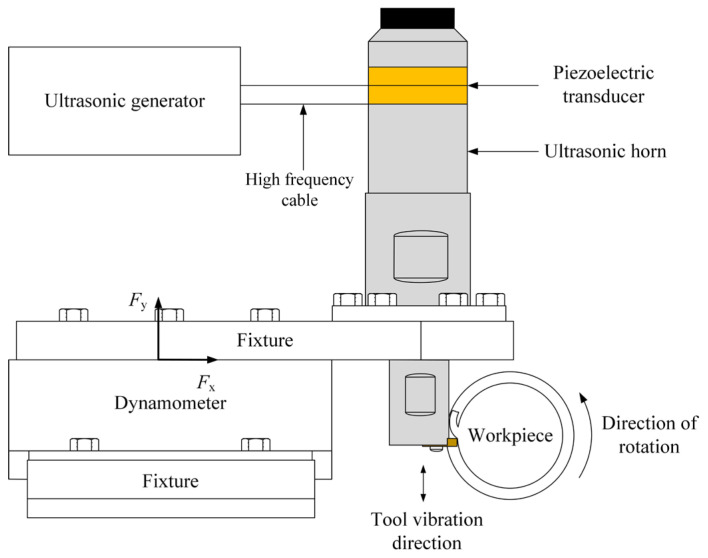
Schematic diagram of ultrasonic vibration system.

The self-developed ultrasonic vibration system is shown in Figure 3. It consists of an ultrasonic signal generator, a transducer, a horn, and a tool. The ultrasonic signal generator generates an ultrasonic frequency high-power electrical signal with adjustable frequency above 18 kHz. The piezoelectric transducer converts the electrical signal into axial mechanical vibration. This high-frequency vibration is transmitted and amplified to the end of the tool through the amplitude amplification of the horn.

**Figure 4:**
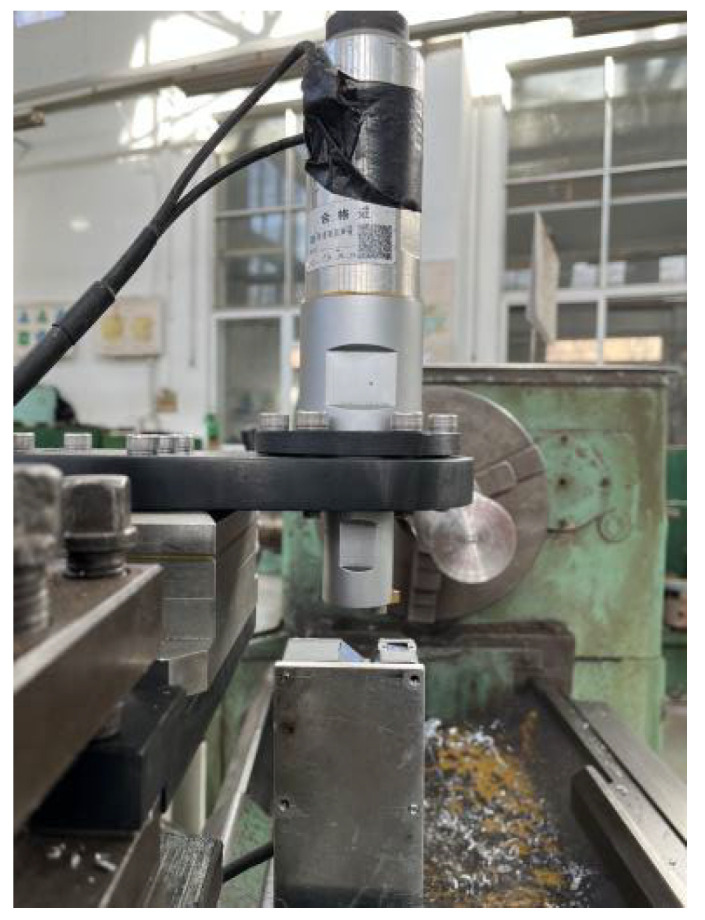
Point laser measurement of ultrasonic amplitude.

The resonant frequency of the ultrasonic vibration system in this experiment is 19.7 kHz. As shown in Figure 4, the laser displacement sensor (LK-H025, KEYENCE, Osaka, Japan) is used to measure the amplitude of the tool before the experiment, and the maximum amplitude can reach 12 μm. The output amplitude can be changed by changing the output power of the power supply.

**Figure 5:**
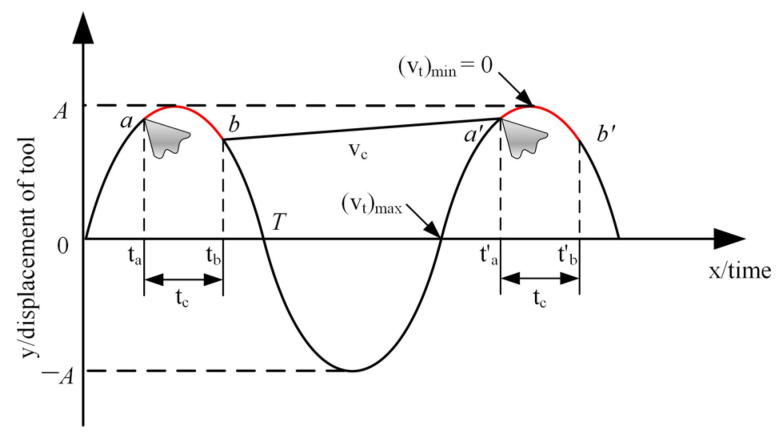
Tool cutting trajectory.

Figure 5 illustrates the path of the cutting tool. The tool of UAT moves along the feed direction while ultrasonic vibration and the trajectory is approximately a sine curve. In the range of red curve, the tool contacts the workpiece for cutting motion.The tool cuts into the workpiece at the time of *t*_a_, and the vibration direction changes after crossing the vertex. When it reaches the time of *t*_b_, the longitudinal velocity of the tool is greater than the rotation speed of the workpiece, and the tool is separated from the workpiece so that the periodic reciprocating realizes the purpose of intermittent cutting.

Critical separation speed (*v*_c_) of the tool and the workpiece rotation speed (*v*) is as follows [26]:

(1) $$v_{c}=2\pi fA,$$

(2) $$v_{}=\pi nD$$

In the formula, *f* is the ultrasonic vibration frequency, *A* is the vibration amplitude, *n* is the workpiece rotation speed, and *D* is the workpiece diameter. According to the existing research, the optimal turning speed should be 1/3 of the critical speed (*v*_c_ = 88 m/min) of the tool. Therefore, the optimal turning speed can be calculated as *v* = 29.3 m/min.

### 2.3. Experimental Methods

The experiment was designed according to the single factor method. Different from CT, UAT is limited by critical speed and other factors. Therefore, on this principle, smaller cutting speed, depth of cut and feed rate were selected in this study, and all process parameters were selected based on UAT literature [17,18,27,28,29]. Referring to existing literature, the cutting speed is 10~100 m/min, the depth of cut is 0.05~0.4 mm, the feed rate is 0.05~0.4 mm/rev and the amplitude is 0~15 μm. Therefore, the parameters selected in this experiment are shown in the Table 3. By changing parameters such as cutting speed, feed rate, cutting depth, and vibration amplitude, the changing law of cutting force and surface roughness with machining parameters was explored, and the machined surface morphology and chip morphology were observed. What’s more, the machinability of UAT of GH4068 superalloy was studied compared with CT, and the cutting length of each parameter group is 20 mm. The study consisted of 27 experiments, each consisting of four cutting parameters. To reduce random errors in the experiment, each experiment was repeated three times. In order to avoid the influence of tool wear on the experimental results, the tool was replaced after every experimental.

In this experiment, a dynamometer (9257B, Kistler Instrumente AG, Winterthur, Switzerland) was used to measure the three-dimensional cutting force. The dynamometer has four 3-component sensors, each with three pairs of quartz plates that convert the forces into electric charges. LN5861 charge amplifier (Jiangsu Lianneng Electronic Technology Co., Ltd, Changzhou, China) was used to convert the charge signal of dynamometer into voltage signal output, and the electric signal was digitized and stored in the computer by a USB-1902 multifunctional data acquisition card (ADLINK TECHNOLOGY, Shanghai, China). Finally, ADLINK U-test software (v2.4.4.0123) was used for data processing. The dynamometer is located between the ultrasonic device and the skateboard to measure the cutting force in real time. The arithmetic square root Ra of the surface contour was used to evaluate the surface machined by different turning methods. Besides, the Tyler Hopson surface profiler (CLI2000, Taylor Hobson Ltd., Leicester, UK) was selected as the measuring instrument. The 3D surface optical profile meter (NewView9000, ZYGO Corporation, Middlefield, CT, USA) was used to observe the two-dimensional and three-dimensional surface morphology of the 800 μm × 800 μm square area of the machined surface. In order to observe the surface morphology and chip morphology of the workpiece, the super depth of a field microscope (OM; VHX-600E, KEYENCY, Osaka, Japan) was used.

## 3. Result and Discussion

### 3.1. Cutting Force

The magnitude of cutting force is considered an important indicator to evaluate the quality of cutting process, which directly affects surface quality, chip formation, and dimensional accuracy [20]. Currently, when processing difficult-to-machine materials such as superalloys, only low removal rates are used to avoid excessive tool wear and reduce cutting force. UAT has demonstrated its enormous advantages [29,30,31]. By changing the cutting speed, feed rate, cutting depth, and vibration amplitude while keeping other parameters constant, the cutting force levels and variation patterns of UAT and CT of GH4068 superalloy are compared. Typically, in turning GH4068 superalloy, a lower feed rate and cutting depth are used. Due to the use of hard alloy tools with a larger tool tip radius in the experiment, the cutting force in the feed direction is much smaller than that in the other two directions, which is ignored in our analysis.

**Figure 6:**
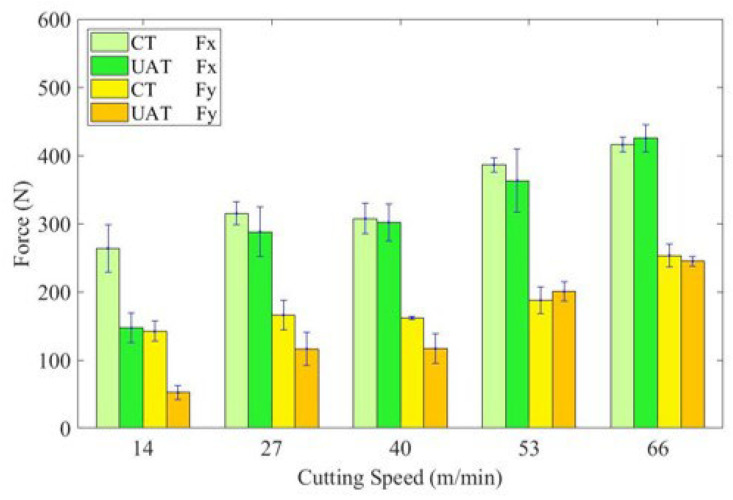
Histogram of the variation of cutting force with cutting speed.

Figure 6 shows the variation of cutting force of UAT and CT of GH4068 superalloy at different cutting speeds. For CT, the cutting force increases with the increase of cutting speed. Due to the good plastic deformation ability of GH4068 superalloy, the actual rake angle of the tool decreases as the cutting speed increases, while the cutting force increases as well. In the UAT experiment, with a cutting speed from 14 to 66 m/min, *F*_x_ increased from 147N to 425N and *F*_y_ increased from 52N to 245N. For UAT, when the cutting speed is small, the cutting force decreases significantly in both the *F*_x_ and *F*_y_ directions. When the cutting speed is 14 m/min, the *F*_x_ and *F*_y_ decrease by approximately 44% and 63%. Nevertheless, as the cutting speed increases, the reduction in cutting force of UAT decreases and gradually approaches that of CT. According to Section 2.1, the cutting speed increases the amount of time the tool spends in contact with the workpiece during UAT. When the cutting speed exceeds the critical speed threshold, the tool cannot be separated from the workpiece, and the cutting force increases.

**Figure 7:**
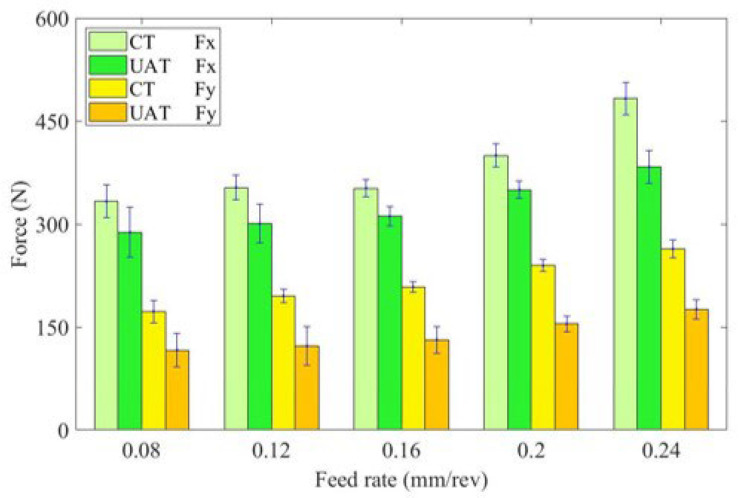
Histogram of cutting force variation with feed rate.

Figure 7 illustrates the variation in cutting force with different feed rates. As the feed rate increases, the cutting force also increases significantly for CT and UAT of GH4068 superalloy. This is because with the increase of feed rate, the actual cutting area of the tool increases. In addition, the friction between the chip and the front edge of the tool increases, and the cutting force increases [32]. The addition of ultrasonic vibration significantly reduces cutting force. Cutting force reductions are most significant at 0.08 mm/rev feed rates, with *F*_x_ and *F*_y_ reduced by approximately 14% and 33%, respectively. A higher feed speed results in an increase in cutting force increases and the load increases, which suppresses ultrasonic vibration and weakens the effect of reducing cutting force. Consequently, ultrasonic vibration has a greater effect at lower feed speeds than at higher feed speeds.

**Figure 8:**
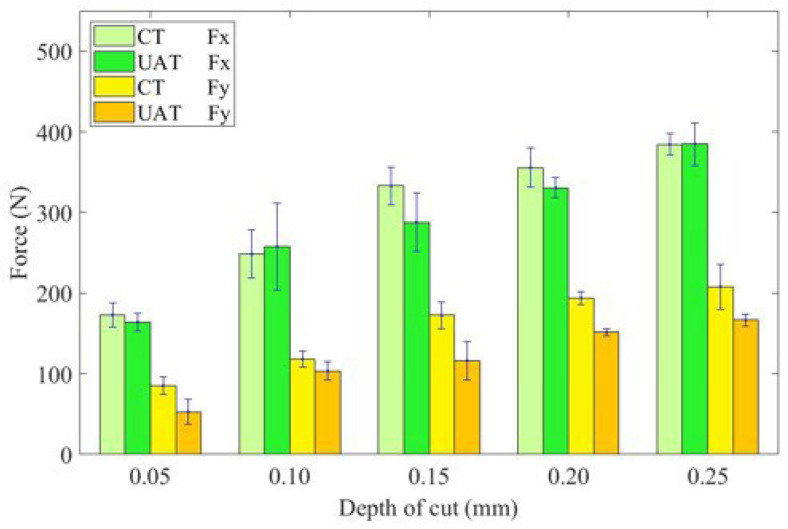
Histogram of the variation of cutting force with cutting depth.

In Figure 8, the change in cutting force increases as cutting depth increases. Under both UAT and CT, the cutting force increases significantly with the cutting depth. Because of the increase in cutting depth, there is an increase in the cutting area and cutting thickness, resulting in an increase in deformation resistance and friction, as well as an increase in cutting force. UAT shows the most significant reduction in cutting force at a cutting depth of 0.15 mm, but the reduction rate of cutting force in UAT is nonlinear relative to CT. It increases from 11.6% at 0.05 mm to 24.7% at 0.15 mm and then decreases to 4.1% at 0.25 mm. 

**Figure 9:**
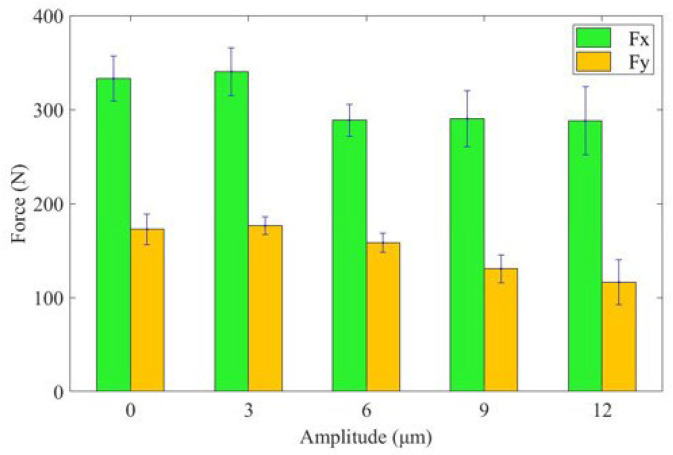
Histogram of cutting force with amplitude change.

Figure 9 illustrates the influence of different amplitudes on cutting force. The histogram shows that with an increase in ultrasonic amplitude, the *F*_x_ and *F*_y_ of UAT first increase, and then decrease. UAT’s cutting force increases slightly when the ultrasonic amplitude reaches 3 μm. When the ultrasonic amplitude is 12 μm, the reduction in cutting force is most significant, with the *F*_x_ and *F*_y_ reduced by 14% and 33%, respectively. This makes the machining effect of UAT better than that of CT.

### 3.2. Surface Roughness

With the frequent starting and stopping of aircraft engines, the pressure they endure constantly changes. Through the analysis of engine parts failure, it is found that poor surface quality of machined parts can lead to faster fatigue damage and accelerate the failure of components. Therefore, controlling the surface quality of parts is also crucial for aircraft engines’ service life and reliability. Surface roughness and surface morphology are important indicators for analyzing surface quality, and proper cutting parameters can improve surface quality [33].

**Figure 10:**
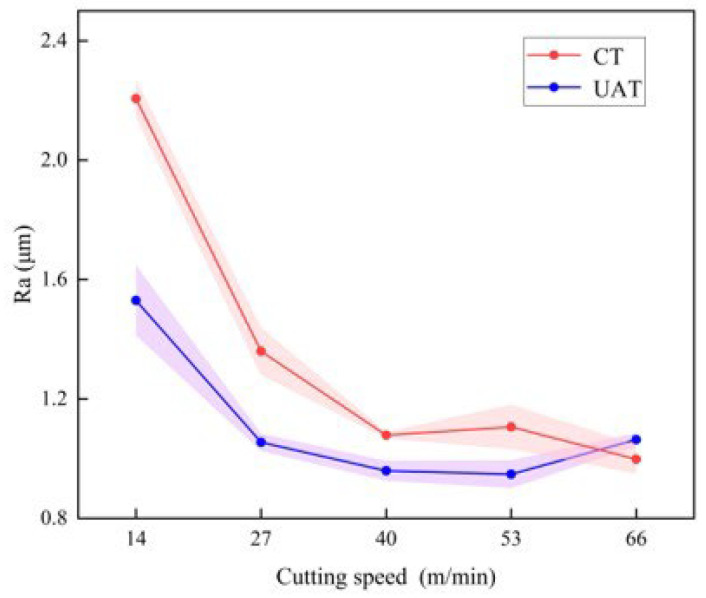
The variation of surface roughness with cutting speed.

The variation of surface roughness with cutting speed is shown in Figure 10. Under the condition that other cutting parameters remain unchanged, the cutting speed is changed, and the GH4068 superalloy turning experiment is carried out. For CT, the cutting force increases overall with cutting speed. This is because cutting speed influences surface roughness mainly caused by physical factors. When the cutting speed is high, plastic deformation is reduced. Since GH4068 superalloy has superior plastic deformation capabilities, cutting speed has a more significant effect on surface roughness. UAT applies vibration in an error-insensitive direction, which reduces the plastic deformation of the workpiece surface and reduces the surface roughness without affecting the geometric accuracy [34]. As shown in the diagram, ultrasonic machining significantly reduces surface roughness at speeds below 66 m/min. When the cutting speed is 14 m/min, the surface roughness after UAT is the most obvious, and Ra is reduced by about 31%. With the increase in cutting speed, the separation between tool and workpiece is weakened, and the improvement effect of UAT on surface roughness is reduced.

**Figure 11:**
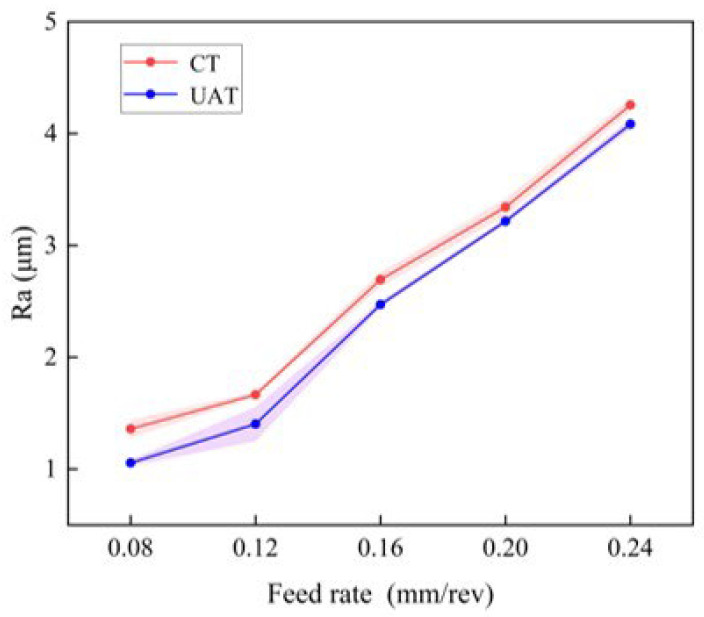
The variation of surface roughness with feed rate.

Figure 11 further explores the effect of feed rate on surface roughness. When the feed rate increases from 0.08 mm/rev to 0.24 mm/rev, UAT surface roughness is the same as that of CT. This is due to the fact that as the feed rate increases, the relative movement speed of the tool and the workpiece increases, and the residual area increases as well. Further, the larger feed will result in an increase in the tool load, which will increase the cutting force, and lead to deterioration of the surface quality. The alternating motion of the tool greatly reduces the surface roughness in UAT. With a feed rate of 0.08 mm/rev, the surface roughness is most evidently reduced, resulting in a reduction of 23% compared to that of ordinary turning. As the feed rate is increased, the reduction rate of Ra under ultrasonic vibration processing decreases from 23% at 0.08 mm/rev to 4% at 0.24 mm/rev. Plastic deformation plays a significant role in surface roughness when the feed rate is low. As a result of the vibration generated by UAT, the plastic deformation of the cutting area is reduced, and the surface roughness is also reduced. Nevertheless, with higher feed rates, geometric factors take precedence, and with higher loads, ultrasonic vibration is less prominent. Thus, UAT’s surface roughness approaches that of CT.

**Figure 12:**
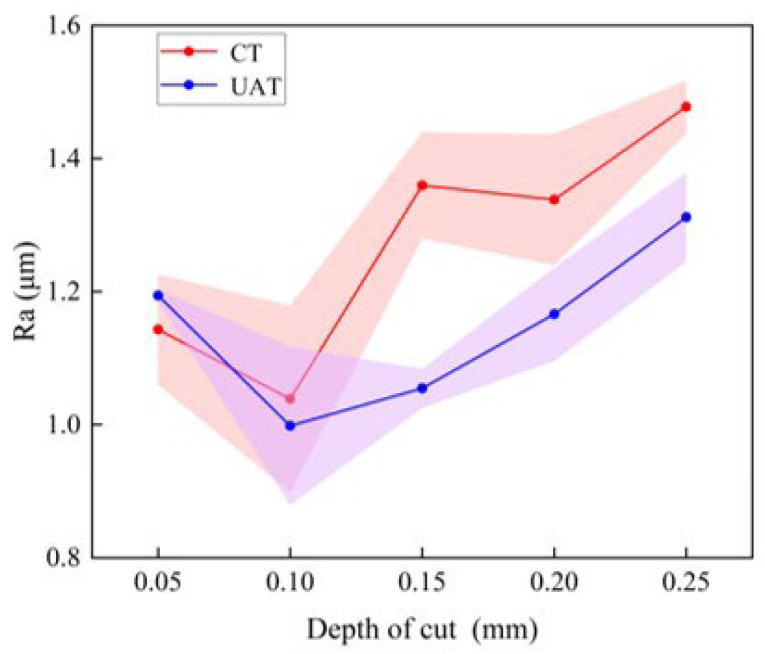
The variation of surface roughness with depth of cut.

The Figure 12 illustrates how surface roughness varies with cutting depth. From the above figure, it is evident that the depth of the cutting has little influence on the roughness of the machined surface. As the cutting depth is changed, the trend of UAT and CT remains the same. Ra decreases with a cutting depth of 0.05 mm to 0.10 mm. Essentially, when the cutting depth is too small, normal cutting is not possible, and the blade slips on the surface of the workpiece, which increases the plastic deformation of the workpiece. As the cutting depth increases from 0.10 mm to 0.30 mm, the surface roughness increases. When the cutting depth is 0.15 mm, the surface roughness of UAT is significantly reduced compared to CT, and Ra is reduced by approximately 23%.

**Figure 13:**
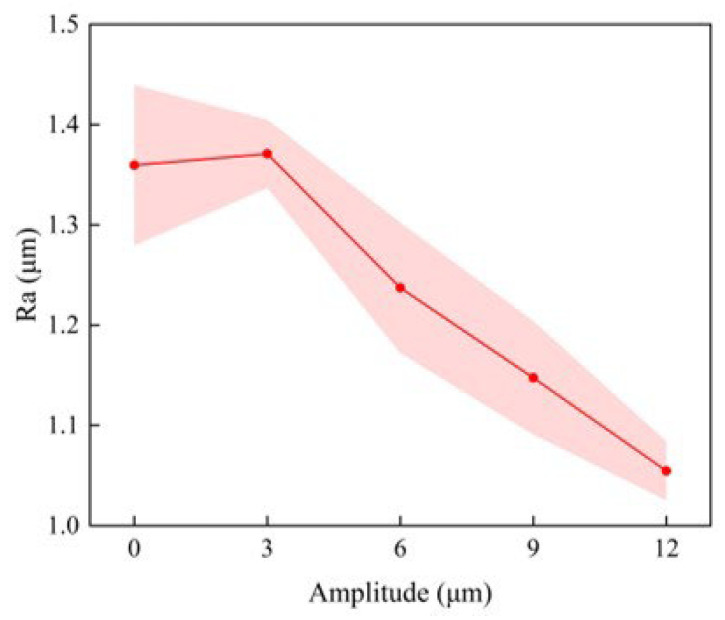
The variation of surface roughness with amplitude.

Furthermore, ultrasonic amplitude has a significant impact on surface roughness in addition to conventional cutting parameters. Figure 13 indicates that when the vibration amplitude is 3 μm, the Ra value of UAT GH4068 is higher than that of CT. We find by calculation that the critical speed of UAT is 22 m/min. Due to the high cutting speed, the tool and the workpiece cannot be separated, and the effect of adding ultrasonic vibration cannot be observed. Furthermore, due to the high relative speed between the tool and workpiece, continuous impact between the tool tip and the blade leads to no significant improvement in surface roughness even when a small amplitude is added. However, as vibration amplitude increases, the tool and workpiece become separated for a longer period of time, and the surface roughness after processing decreases significantly. There is a significant decrease in surface roughness when the amplitude is 12 μm, indicating that a larger amplitude can result in a better surface quality.

### 3.3. Surface Morphology

The surface morphology of the workpiece is the key to evaluating the processing quality [35]. According to the analysis of Section 2.1 and Section 2.2, the following cutting parameters significantly improve the surface quality of ultrasonic machining: cutting speed 27 m/min, feed speed 0.08 mm/rev, cutting depth 0.15 mm, and UAT vibration amplitude 12 μm. Surface morphology characteristics of UAT and CT workpieces are compared under the same cutting parameters.

**Figure 14:**
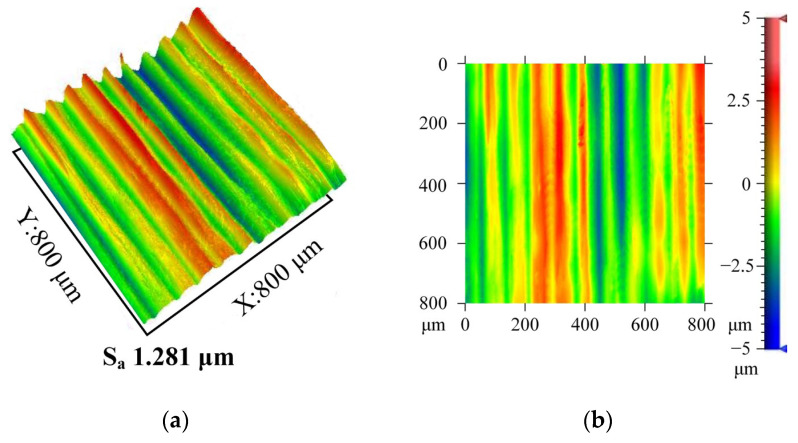

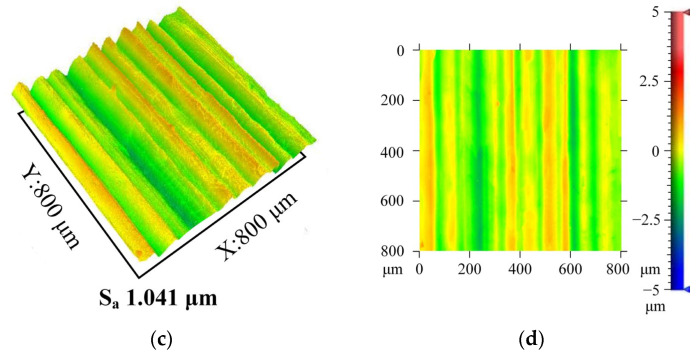
Surface morphology: (**a**) 3D morphology during CT, (**b**) 2D morphology during CT, (**c**) 3D morphology during UAT, (**d**) 2D morphology during UAT.

In Figure 14, the typical surface morphology characteristics of UAT and CT are compared. Sa was used to evaluate the three-dimensional surface roughness. Compared to CT, UAT’s three-dimensional surface roughness decreased by about 19%, indicating that UAT’s average roughness decreased within the sampling area. CT shows an obvious periodicity, with the contour peaks separated by approximately 80 mm, indicating a feed rate of 0.08 mm per revolution. On the machined surface obtained by UAT, the periodicity of this surface profile is weaker. A smoother surface is obtained by alternating the motion of the tool in UAT. When the tool and workpiece are turned, the radial force will cause a relative displacement of the tool and workpiece. Surface quality and tool stability are directly related to the radial force. The smaller the radial force, the better the stability between the tool and the workpiece. Based on the analysis of cutting force in Section 2.1, a smaller radial force is also one of the key factors for UAT to achieve a better surface.

**Figure 15:**
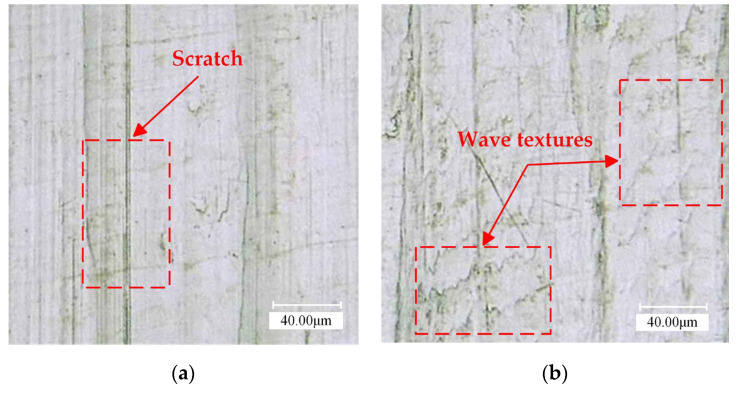
Microscopic images of machined surfaces: (**a**) amplified 500 times in CT; (**b**) amplified 500 times in UAT.

Figure 15 shows the surface morphology of UAT and CT GH4068 superalloy under 500 times magnification via super depth of field. Stripes are distributed evenly on the machined surface of CT. However, different scratches have different lengths, and the differences are obvious. Apart from the tool marks, pits are also left on the workpiece as a result of the tool. For UAT, the surface texture is flat, and the ultrasonic vibration leaves textures on the surface. However, despite the complex surface texture, the tool marks are uniform in size and spacing, and the surface is smoother and more delicate in appearance.

### 3.4. Chip Morphology

The process of turning superalloys is essentially the process of material removal to form chips. In the machining process, the geometric shape of chips can reflect friction between the tool and the workpiece because the shape of chips is closely related to cutting force and cutting heat. Thus, it is necessary to judge machinability based on the appearance of the chips [36]. The chip morphology characteristics of UAT and CT were observed under the same cutting parameters of cutting speed 27 m/min, feed speed 0.08 mm/rev, cutting depth 0.15 mm, and UAT vibration amplitude 12 μm.

**Figure 16:**
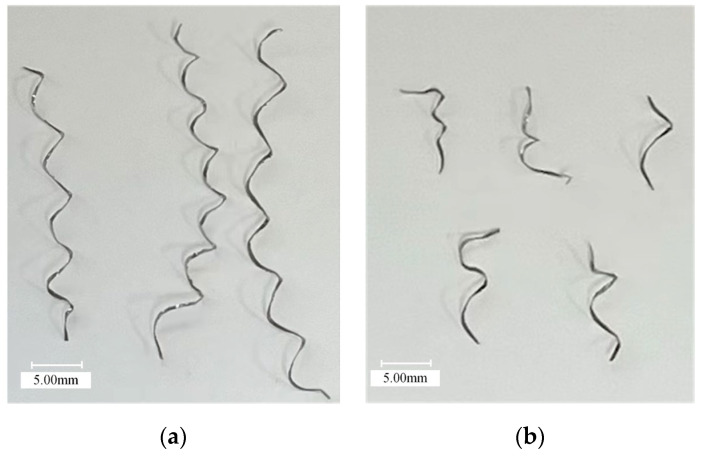
Chip morphology: (**a**) CT and (**b**) UAT.

Figure 16 shows the chip photos of UAT and CT GH4068 superalloy. It is evident that UAT and CT are spiral chips, but there are obvious differences between them. The chip of UAT is short, bending radius is about 2mm, the chip-breaking effect is evident, and it is loosely spiraled. CT exhibits a high degree of chip curling. The chip bending radius is about 4 mm, which provides certain toughness and is not prone to chip fractures.

**Figure 17:**
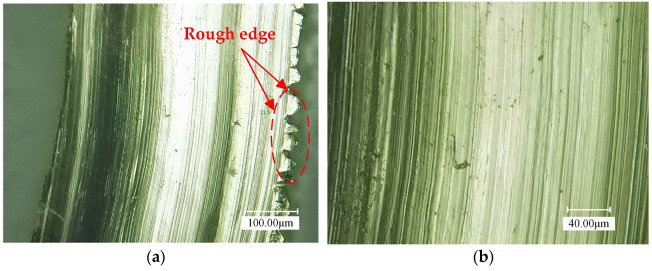

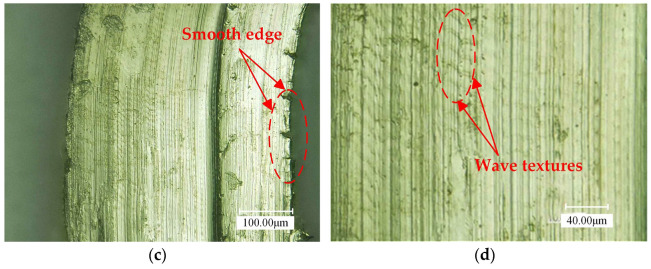
Chip micro morphology: (**a**) amplify 500 times in CT; (**b**) amplify 1000 times in CT; (**c**) amplify 500 times in UAT; (**d**) amplify 1000 times in UAT.

Figure 17 illustrates the chip morphology observed by the ultra-depth-of-field microscope. The chips of UAT and CT are enlarged by 500 times and 1000 times, respectively. There are many sharp sawteeth formed by a chipped edge of CT, which is large and sharp, making it easy to scratch the machined surface and affecting the quality of the machining. Additionally, the chip surface contains cracks, scratches, depressions, and other defects. Ultrasonic vibration reduces the burr phenomenon at the edge of the chip, forming a rectangular sawtooth with a relatively smooth finish, and there is no significant difference between the sawtooth, which is evenly distributed. Meanwhile, the chip surface is smooth, delicate, and free of obvious defects, and ripples generated by ultrasonic vibration can be observed.

## 4. Conclusions

This paper presents the results of single-factor experiments conducted on the CT and UAT GH4068 superalloys. In this study, the effects of cutting speed, feed speed, cutting depth, and vibration amplitude were examined on cutting force and surface roughness. Several parameters were examined in order to determine the surface morphology and chip morphology of the workpiece. Based on the experimental results, the following conclusions were reached:

1. Choosing the appropriate process parameters of UAT can reduce the cutting force of GH4068 superalloy. When the cutting speed is 14–53 m/min, the feed speed is 0.08–0.24 mm/rev, and the cutting depth is 0.10–0.25 mm, the cutting force decreases significantly. Compared with CT, the *F*_x_ and *F*_y_ are reduced by a maximum of 44% and 63%, respectively. The reduction of cutting force decreases as process parameters are increased, but UAT still has a significant impact on reducing cutting force.
2. When the cutting speed is lower than the critical speed, UAT has a significant effect on reducing the surface roughness, when the cutting speed is 14–53 m/min, the feed speed is 0.08–0.24 mm/rev, the cutting depth is 0.10–0.25 mm, the effect of UAT is obvious, reducing surface roughness by a maximum of 31%.
3. When using UAT and CT GH4068 superalloy, the surface morphology is obviously different. CT’s surface profile exhibits obvious periodicity. Scratches of different sizes are visible on the machined surface. UAT obtains a smoother surface, and the ultrasonic vibration produces complex ripples, and the tool marks are uniform in size and distribution.
4. The UAT chip is short, the chip-breaking effect is obvious, and the edge of the chip forms relatively smooth rectangular sawteeth. The chip surface is smooth and delicate. For CT, the chip bending radius is large, and the edge of the chip formed many sharp sawteeth.

## Figures and Tables

**Table 1:** Geometrical parameters of tool.

Project	Content
Insert Part Number	WNMG080404N-TF
Nose angle (°)	55
Nose radius (mm)	0.4
Rake angle (°)	7
Rear angle (°)	0

**Table 2:** Chemical composition of GH4068 superalloy.

Elements	Al + Ti	Co	W	Cr	Mo	Ni
Weight %	7.83	24.83	1.19	13.85	2.79	Bal.

**Table 3:** Experimental design.

Exp. No	Cutting Speed (m/min)	Feed Rate (mm/rev)	Depth of Cut (mm)	Vibration Amplitude (μm)
1	14, 27, 40, 53, 66	0.08	0.15	12, -
2	27	0.08, 0.12, 0.16, 0.20, 0.24	0.15	12, -
3	27	0.08	0.05, 0.1, 0.15, 0.20, 0.25	12, -
4	27	0.08	0.15	-, 3, 6, 9, 12

## Data Availability

Not applicable.
